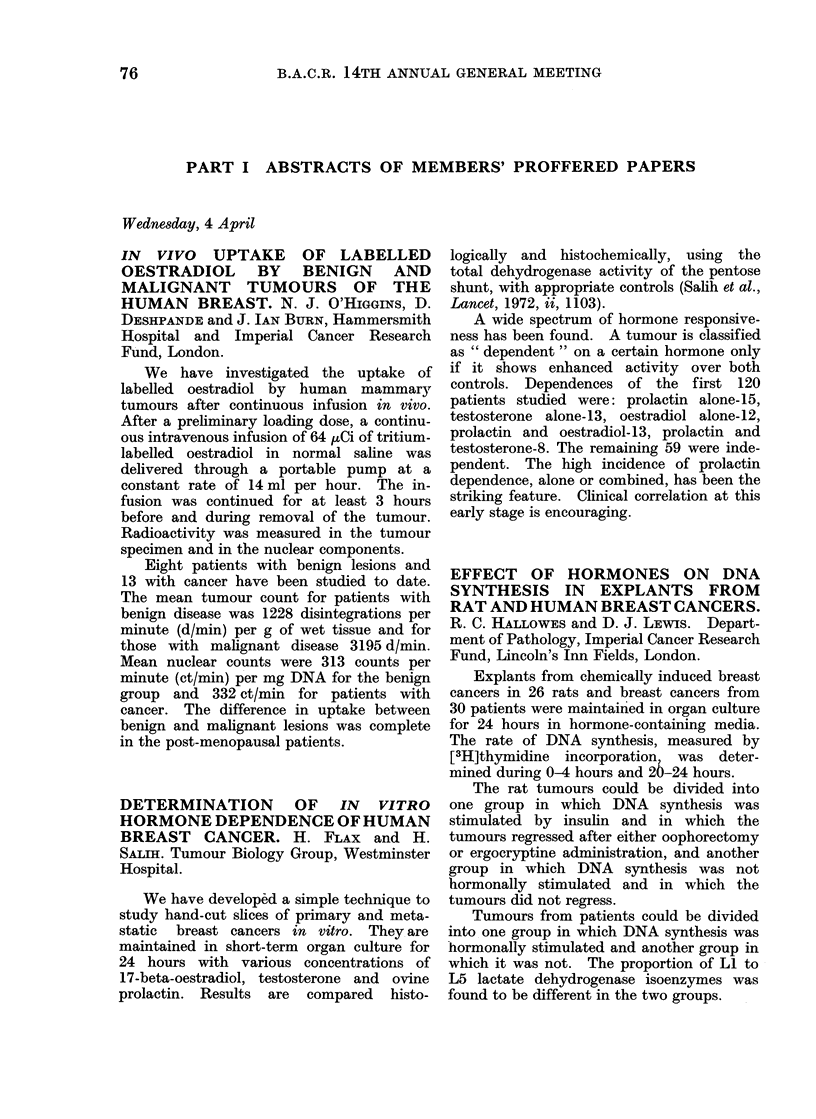# Effect of hormones on DNA synthesis in explants from rat and human breast cancers.

**DOI:** 10.1038/bjc.1973.75

**Published:** 1973-07

**Authors:** R. C. Hallowes, D. J. Lewis


### EFFECT OF HORMONES ON DNA SYNTHESIS IN EXPLANTS FROM RAT AND HUMAN BREAST CANCERS.

R. C. Hallowes and D. J. Lewis. Department of Pathology, Imperial Cancer Research Fund, Lincoln's Inn Fields, London.

Explants from chemically induced breast cancers in 26 rats and breast cancers from 30 patients were maintained in organ culture for 24 hours in hormone-containing media. The rate of DNA synthesis, measured by [$^3$H]thymidine incorporation, was determined during 0–4 hours and 20–24 hours.

The rat tumours could be divided into one group in which DNA synthesis was stimulated by insulin and in which the tumours regressed after either oophorectomy or ergocryptine administration, and another group in which DNA synthesis was not hormonally stimulated and in which the tumours did not regress.

Tumours from patients could be divided into one group in which DNA synthesis was hormonally stimulated and another group in which it was not. The proportion of L1 to L5 lactate dehydrogenase isoenzymes was found to be different in the two groups.